# “False” Ligaments: A Review of Anatomy, Potential Function, and Pathology

**DOI:** 10.7759/cureus.1853

**Published:** 2017-11-16

**Authors:** Marc Vetter, Rod J Oskouian, R. Shane Tubbs

**Affiliations:** 1 Seattle Science Foundation; 2 Neurosurgery, Complex Spine, Swedish Neuroscience Institute; 3 Neurosurgery, Seattle Science Foundation

**Keywords:** suprascapular, mamillo-accessory, transforaminal, transverse, transverse occipital, humeral, ulnar, intrinsic, false ligaments

## Abstract

This paper, although not an exhaustive review of "false" ligaments in the body, describes eight such ligaments. False ligaments are defined as ligamentous structures connecting separate parts of the same bone and are thus immobile. The ligaments reviewed include the suprascapular ligament, the transforaminal lumbar ligaments, the mamillo-accessory ligament, the transverse atlantal ligament, the transverse occipital ligament, the transverse humeral ligament, the coracoacromial ligament, and the transverse part of the ulnar collateral ligament. In this review, the anatomy and histological characteristics of each ligament are reviewed. Furthermore, possible functions and associated pathologies are described.

## Introduction and background

In recent literature, the need for a new set of terminology to describe so-called “false ligaments” has been highlighted by a growing number of authors [1]. For the purposes of this review, the term “false ligament” is used to describe ligaments which do not connect two different bones but rather span two parts of the same bone. The anatomical relevance and function of many of these ligaments remain in question. Furthermore, there is disagreement among researchers as to whether certain false ligaments, such as the transverse humeral ligament, are in fact distinct anatomical structures [2]. A comprehensive investigation into the histology and function of these ligaments, as well as the common pathologies which affect them, will help to more clearly define which ligaments can be described as “false.” This will also allow surgeons and other medical health professionals to better weigh the cost, if any, of compromising or damaging these anatomical structures during procedures [1]. Herein, we review the extant medical literature regarding eight of the “false ligaments” found in the human body: the suprascapular, transforaminal lumbar, transverse atlantal, mamillo-accessory, transverse occipital, and the transverse part of the humeral, coracoacromial, and transverse ulnar collateral ligaments.

## Review

Suprascapular ligament

**Figure 1:**
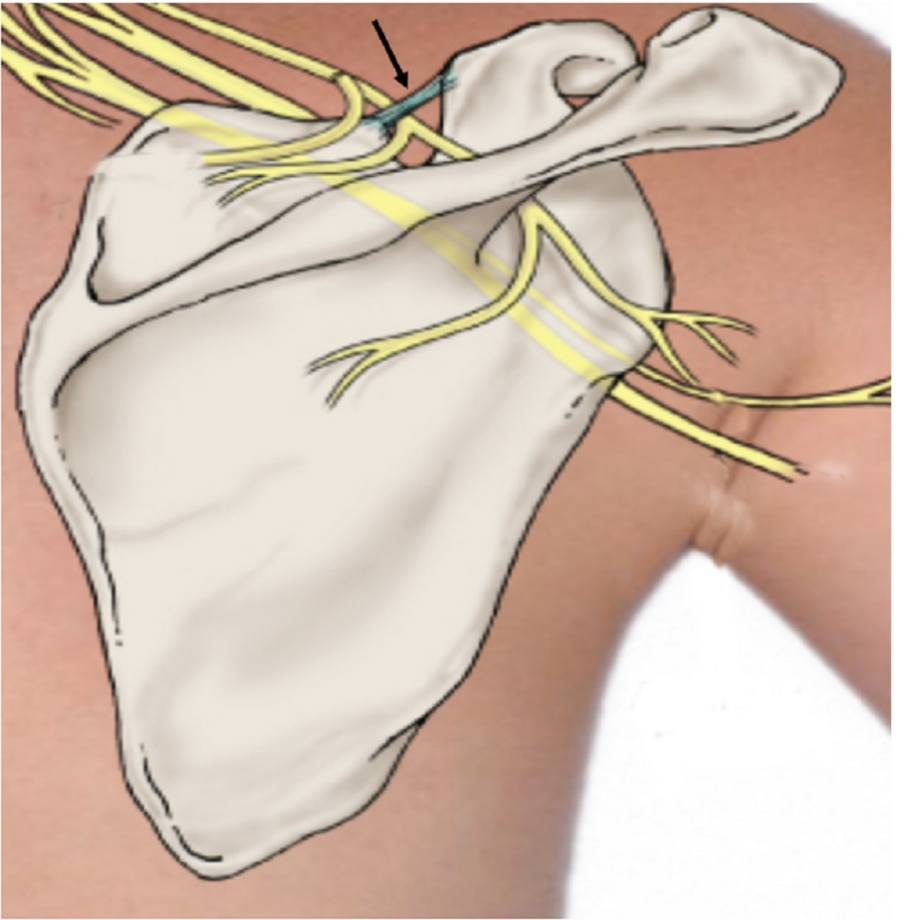
Schematic drawing of the posterior scapula illustrating the suprascapular ligament (arrow)

The suprascapular ligament (SSL) (Figure 1), also known as the superior transverse scapular ligament, spans the distance between the base of the coracoid process and the medial ridge of the suprascapular notch. As it passes over the suprascapular notch, it forms the suprascapular foramen [3]. The suprascapular nerve, which rises from the upper trunk of the brachial plexus, usually runs through this foramen. Although there is little research exploring the potential stabilizing properties of this ligament, the SSL has been the object of increased study due to its role in the causation of suprascapular nerve entrapment syndrome (SNES) [4]. This syndrome, characterized by compression of the suprascapular nerve as it runs through the suprascapular foramen, can, in many cases, lead to paralysis of the infraspinatus and supraspinatus muscles. Several characteristics of the SSL, including the extent to which it is ossified, directly affect the likelihood of developing SNES. Individuals with an ossified SSL are much more likely to suffer from SNES. A study by Tubbs, et al. suggested that the rate of SSL ossification in the general population is around 5% [3]. Rates of SSL ossification differ by sex, with the ligament more likely to ossify in men [4]. In order to reduce the pain caused by SNES, surgeons have advocated for releasing the SSL when impingement occurs. Decompression of the subscapular nerve is most often performed by releasing the SSL at the suprascapular notch using an open posterior approach [5]. Although the structural importance of the SSL remains an area in need of further investigation, its relationship to the development of SNES has been the subject of considerable research.

Transforaminal ligaments

**Figure 2:**
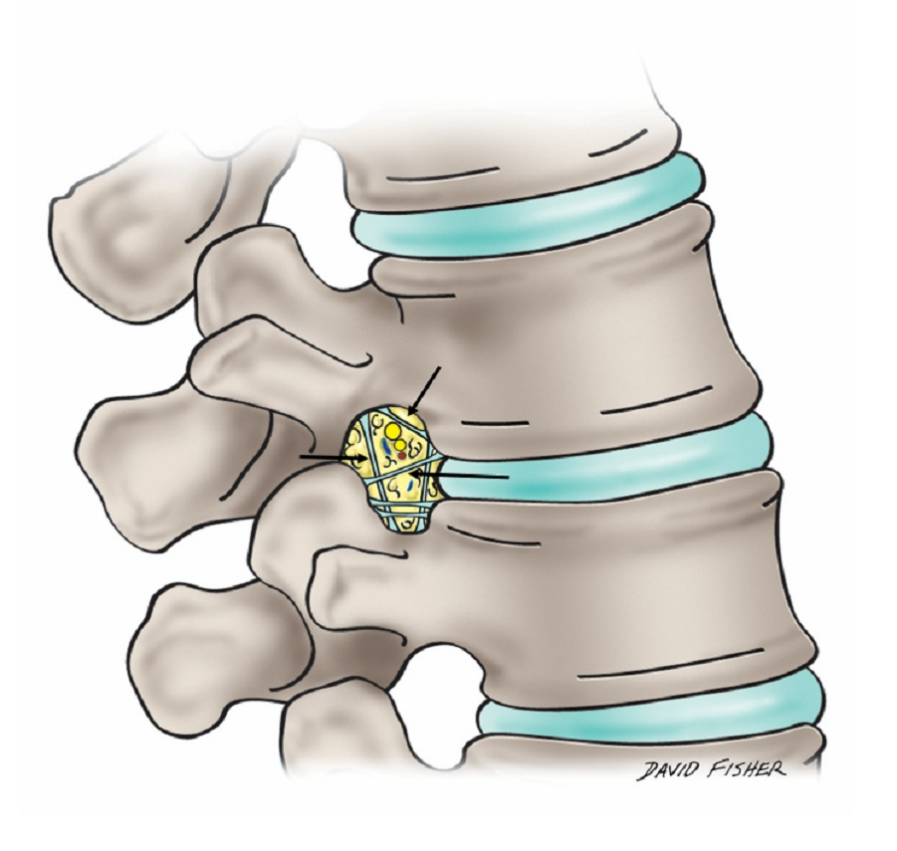
Schematic drawing illustrating the transforaminal ligaments (arrows) of the lumbar region

The transforaminal ligaments (TFLs) (Figure 2), first illustrated by the French anatomist Bougery in 1832, were long thought to be pathological or anomalous structures in the intervertebral foramina of the lumbar spinal column [6]. Five distinct types of TFLs have been described: the superior and inferior transforaminal ligaments, the superior and inferior corporotransverse ligaments, and the mid-transforaminal ligaments. Together, these ligaments compartmentalize the intervertebral foramina, with spinal nerves, lymphatics, and vascular tissue occupying separate spaces within the compartments [6]. A study by Min, et al. suggests that TFLs are regularly present in lumbar vertebrae - observable in approximately 80% of lumbar intervertebral foramina [7].

Although TFLs are no longer considered anomalous, their anatomical function is still contested. It has been theorized that the space taken up by the TFLs in the intervertebral foramina leads to lumbar stenosis, compressing the nerves and vessels in the foramina, and causing radiating back pain [7]. However, more recently, Zhao, et al. have proposed that the incidence of intervertebral foraminal stenosis due to TFL compression is relatively rare. Furthermore, TFLs may serve to protect the nerve roots exiting the lumbar vertebrae [8]. The ambiguity surrounding the anatomical significance of TFLs warrants further research into the histological and functional characteristics of these ligaments.

Mamillo-accessory ligament

**Figure 3:**
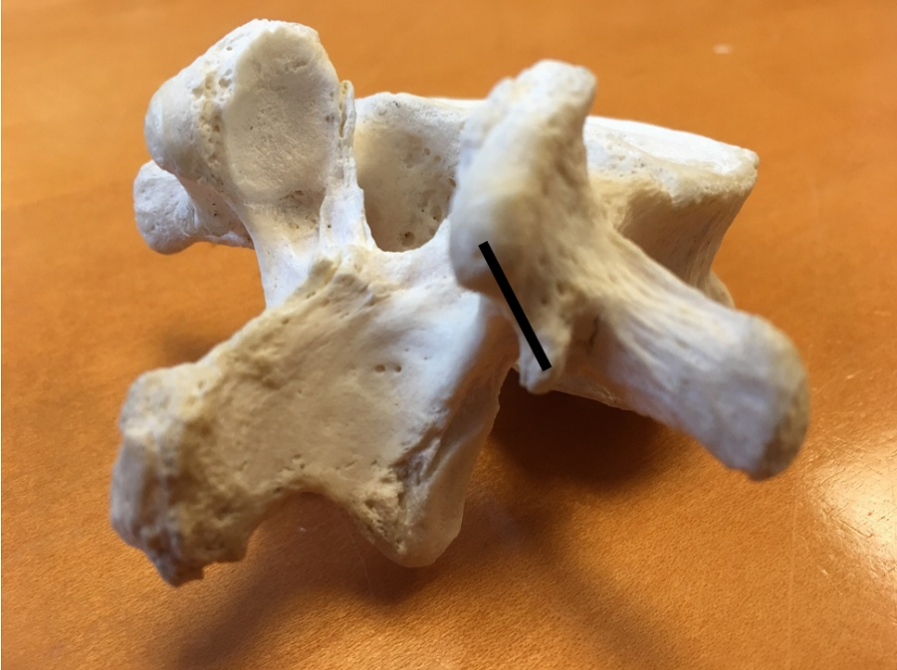
Lumbar vertebra with black line representing the mamillo-accessory ligament

The mamillo-accessory ligament (MAL) (Figure 3) extends between the posterior aspect of the mammillary process and the ipsilateral accessory process on each side of the lumbar vertebrae. A fibrous band approximately 1-2 mm thick, it forms a foramen by covering a notch lying in between the accessory and mammillary processes [9]. The resultant foramen covers a section of the medial branch of the dorsal ramus. The MAL is prone to ossification, particularly in the lower lumbar vertebrae. A study by Bogduk, et al. suggested that approximately 11% of MALs on the L5 vertebra showed partial to complete ossification of the ligament with a resultant formation of a foramen [9].  

In the context of the musculoskeletal system, the MAL lies between the longissimus thoracis and the multifidus muscles. It has been posited that the MAL may, in fact, be an extension of the longissimus thoracis tendon rather than an independent ligamentous structure. Bogduk, et al., who first proposed a name for the MAL, noted that the transverso-articulaire ligaments, located in the cervical region of the spine, share a similar length and structure to the MAL. Furthermore, when present, the transverso-articulaire ligaments also cover the medial branch of the dorsal ramus. Upon histological analysis, it has been determined that the transverso-articulaire ligaments are in fact continuations of the tendon in the semispinalis capitis, calling into question whether or not the structurally related MALs are distinct ligamentous structures or an extension of muscle in the lumbar region. [9]

The ossification of the MAL, unlike that of the suprascapular ligament, is unlikely to be the cause of symptomatic nerve compression. Lynton, et al. have reported findings which demonstrate that the medial branch of the dorsal ramus accounts for a largely insignificant portion of the foramen created by the MAL [10]. Therefore, as there are very little pathologies associated with the MAL, its purpose and function have yet to be thoroughly investigated. Although it has been suggested that the function of the MAL, especially when ossified, is to partially protect the medial branch of the dorsal ramus nerve, the histological characteristics and processes which lead to the ossification of the MAL are largely unknown [9].

Transverse atlantal ligament

**Figure 4:**
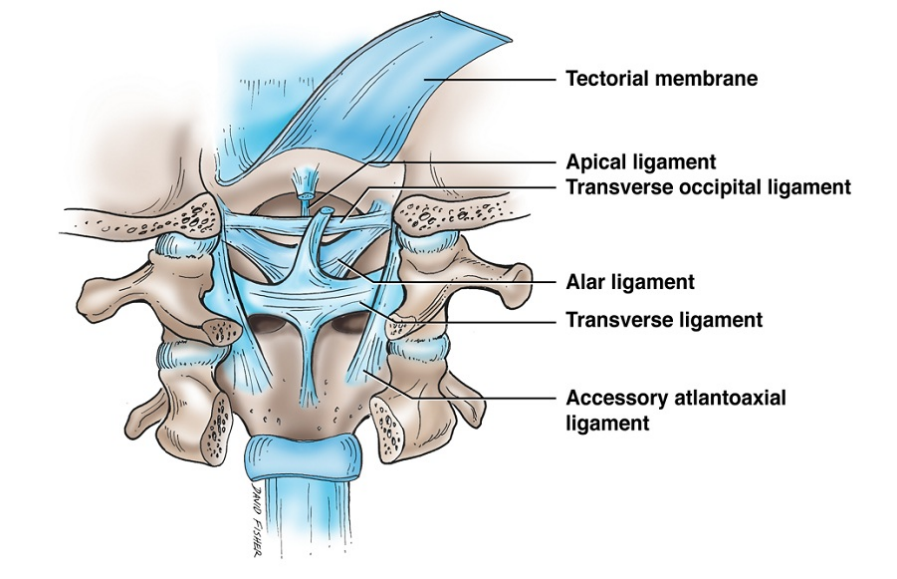
Schematic drawing of the ligaments of the craniocervical junction Note the transverse ligament connecting the atlas from one side to the other.

The transverse atlantal ligament (TAL) is one of the most biomechanically critical structures of the craniocervical junction (Figure 4) [11]. The TAL is present as a wide, strong band stretching across the atlantal ring immediately posterior to the dens [12]. About 20 mm in length, it attaches on the medial side of either lateral mass in the atlas. Extending from the center of the TAL, longitudinal bands descend to the posterior side of the axis and ascend to the occipital bone. Together, this ligamentous complex is called the cruciate ligament [12]. Histologically, the area from which the superior and inferior longitudinal bands of the TAL extend contains dense collagen fibers which mesh together at a variety of different angles, creating a strong interwoven structure.

As a primary stabilizing ligament, the TAL is at risk of being torn or disrupted when the atlas is fractured. As atlas fractures are a relatively common occurrence, accounting for approximately 3-13% of all cervical spine injuries, knowledge of the impact of such an injury on the TAL is important [13]. In the event of an atlas fracture, the structural integrity of the TAL is often used to classify the injury into one of two major subsections: stable or unstable. If the TAL is significantly compromised, the injury is classified as unstable and usually requires surgery [14]. Debernardi, et al. reported that TAL injuries themselves often are categorized into two types depending on the origin of the injury. Type I TAL injuries are the result of disruption to the ligamentous substance itself, whereas type II injuries occur when the ligament avulses from its insertion site on the lateral mass of C1 [15]. Understanding the causes and classification of various TAL injuries is critical as this ligament, when torn, is incapable of self-repair [11]. Apart from traumatic injury, ossification of the TAL has been reported by Shoda, et al. but is considered an uncommon pathological condition [16].       

Transverse occipital ligament

The transverse occipital ligament (TOL) forms a part of the ligamentous complex that binds the craniocervical junction (CCJ) (Figure 4). It lies posterosuperior to the dens, parallel to the transverse portion of the cruciate ligament [17]. Although the ligaments found at the CCJ have in general been the subject of comprehensive research, the functional significance of the TOL has largely been omitted from these investigations. As a result, the incidence rate of the TOL in the general population varies widely in the literature. In a study of the CCJ, Dvorak, et al. reported a 10% prevalence rate of the TOL [18]. However, later research by Tubbs, et al. reported nearly an 80% rate [17]. The TOL lies immediately superior to the alar ligament; therefore, it has been proposed that the TOL serves a similar role as the alar ligament, namely providing additional resistance of axial rotation and lateral flexion [19-20].

Transverse humeral ligament

**Figure 5:**
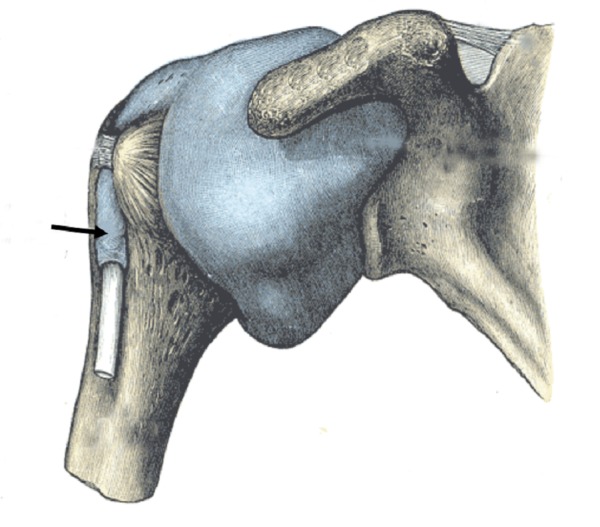
Depiction of the transverse humeral ligament (arrow) From Gray's Anatomy (1858, public domain)

The transverse humeral ligament (THL) originates from the tendon of the subscapularis, which inserts onto the lesser tubercle of the humerus (Figure 5). From the lesser tubercle, the THL extends laterally across the intertubercular sulcus, eventually terminating at the greater tubercle [2]. As it extends over the intertubercular sulcus, it forms a canal, overlaying the tendon of the long head of the biceps brachii muscle. It has been suggested that the anatomical function of the THL may be to act as a retinaculum for this tendon, thereby stabilizing it [12]. Since being first described in 1889 by Scottish anatomist Charles Gordon Brodie, the THL has been described in many anatomical textbooks as a distinct anatomical structure. However, research in the past decade has called into question whether or not the THL should be considered a ligament.

In a study documenting the prevalence of the THL, MacDonald, et al. found that in only 8% of cases did the tendon of the subscapularis insert exclusively onto the lesser tubercle of the humerus. In the majority of cases, the tendon of the subscapularis extended over the intertubercular sulcus, spanning the distance usually covered by the THL. Furthermore, Gleason, et al. demonstrated that through a histological analysis of fibrous extensions covering the intertubercular sulcus, the tissue was more similar to that found in tendinous structures. Elastin, which is usually associated with ligamentous tissue and is minimally present in tendons, was absent in nearly all samples, providing further support to the theory that the THL is, in fact, a continuation of the subscapularis tendon [21].

Transverse ulnar collateral ligament

**Figure 6:**
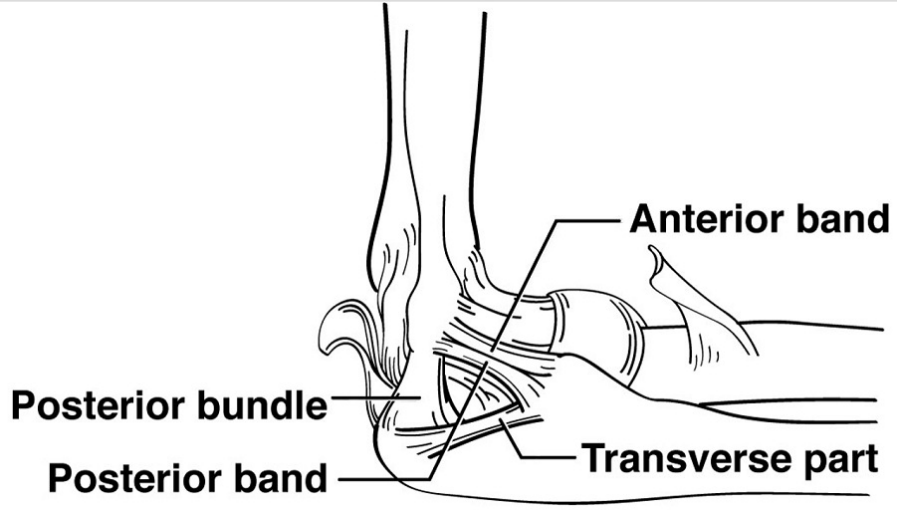
Schematic drawing of the ligaments in the medial elbow joint Note the bands of the ulnar collateral ligament and that the transverse part is limited to the ulna.

The ulnar collateral ligament (UCL), is comprised of three different parts - anterior, posterior, and transverse bands (Figure 6). Only the transverse part of the UCL can be considered a "false" ligament. This ligament has been the subject of a wide variety of studies due to its biomechanical importance in a number of throwing motions associated with sports, such as baseball [22]. However, although the stabilizing effects of the anterior and posterior UCL bands on the elbow joint are relatively well known, the anatomical function of the transverse band is uncertain [23]. The transverse band of the UCL, or Cooper’s ligament, extends medially between the coronoid process of the ulna and the olecranon (Figure 6). The transverse band is limited to the ulna, and thus the structural purpose of the ligament is more difficult to understand than those of the anterior or posterior bands. One proposed function for Cooper's ligament is that it may help the humeral trochlea fit into the trochlear notch [22]. More research to support this hypothesis is necessary. Fuss, et al. argue that it may be of value to study whether or not Cooper’s ligament aids the elbow joint in withstanding valgus stress. If no significant structural purpose for Cooper’s ligament can be determined, it may be of use to define it separately from the rest of the UCL in the anatomical literature [23].

Coracoacromial ligament

**Figure 7:**
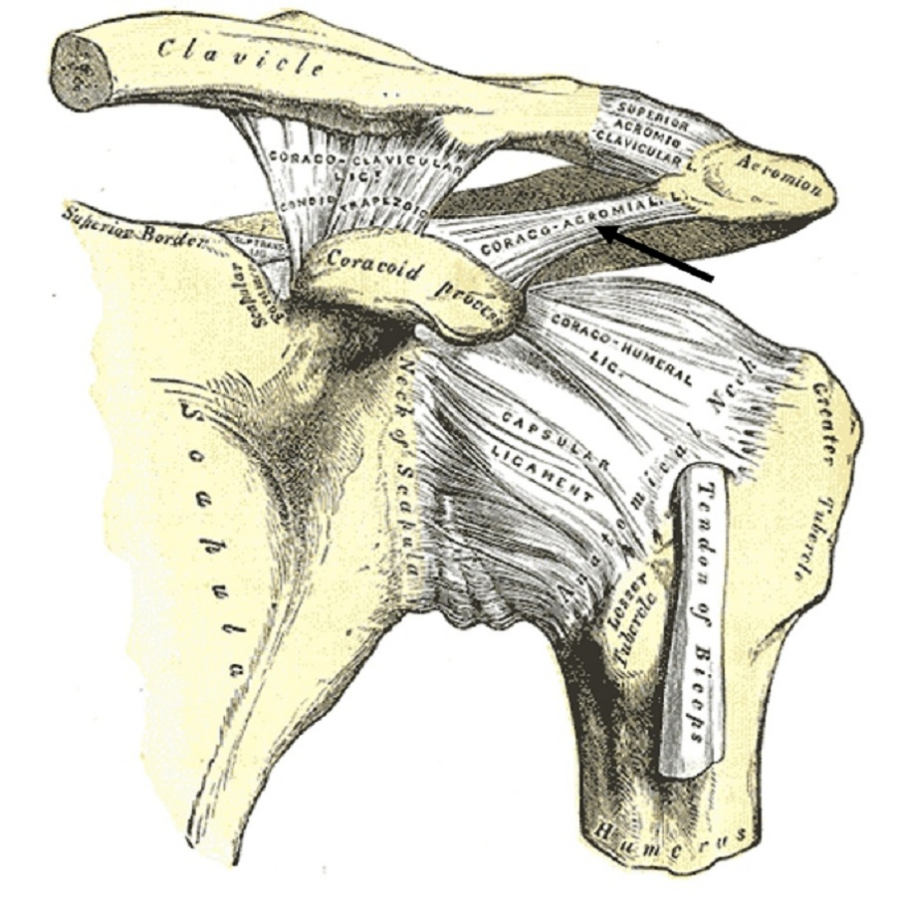
Anterior view of the left shoulder joint Note the coracoacromial ligament (arrow) spanning two parts of the scapula, the coracoid process, and acromion. (From Gray's Anatomy, 1858, public domain).

The coracoacromial ligament (CAL) connects two different parts of the scapula: the coracoid process and the acromion (Figure 7). It extends from the inferior anterolateral surface of the acromion to insert onto the lateral edge of the coracoid process [24]. The CAL forms the coracoacromial arch, which serves several anatomical purposes. First, it contributes to glenohumeral joint stability by mitigating the potential for superior displacement of the humeral head. Second, it helps to transmit mechanical forces exerted on the acromion by surrounding muscles. Finally, the CAL might serve a sensory role. Rothenburg, et al. found that the CAL is innervated by the suprascapular nerve at the ligament’s entheses. This complex innervation includes a relatively high density of Ruffini and Pacinian corpuscles, which signifies that the CAL may be responsible for sending afferent proprioceptive information to the central nervous system, thereby contributing to coordinated shoulder movement.

When ossified or calcified, the CAL can contribute to shoulder impingement syndrome (SIS), which occurs as a result of repetitive contact between the coracoacromial arch and rotator cuff muscles, leading to inflammation [24]. According to Kijima, et al., as the CAL becomes stiffer and more ossified, the pressure exerted by contact between the rotator cuff and the coracoacromial arch increases, aggravating the condition [25]. Histologically, the CAL is a highly fibrocartilaginous ligament with soft collagen bundles running parallel. However, both age and repetitive motion exerting compressive forces on the CAL can increase the rate of calcification and ossification, ultimately increasing the density of fibrocartilaginous tissue. This phenomenon aggravates SIS by intensifying the degenerative contact between the coracoacromial arch and the rotator cuff. Acromioplasty is a surgical procedure commonly used to relieve pressure near the coracoacromial arch, although debate regarding whether or not the CAL should be released without repair during this procedure is still not settled. Some research suggests that releasing this ligament allows increased translation of the glenohumeral joint [26].

## Conclusions

The anatomical function and importance of many of the so-called “false” ligaments require more research in order to be better elucidated. These structures, defined as ligaments connecting different features on the same bone, would benefit from a new system of nomenclature grouping them together. As it stands, the term “false” is frequently applied to refer to these ligaments. However, the term is of little benefit in helping surgeons, clinicians, and researchers to understand both the variation and similarity that exists between such ligaments. Furthermore, it may inaccurately imply that these ligaments universally lack functional importance. We propose using the term “intrinsic ligament” in describing these structures.
